# Applying a biopsychosocial model to migraine: rationale and clinical implications

**DOI:** 10.1186/s10194-022-01471-3

**Published:** 2022-08-11

**Authors:** Chiara Rosignoli, Raffaele Ornello, Agnese Onofri, Valeria Caponnetto, Licia Grazzi, Alberto Raggi, Matilde Leonardi, Simona Sacco

**Affiliations:** 1 Department of Biotechnological and Applied Clinical Sciences, University of L’Aquila, L’Aquila, Italy; 2 Neuroalgology Unit and Headache Centre, Fondazione IRCCS Istituto Neurologico Carlo Besta, Milan, Italy; 3 Neurology, Public Health and Disability Unit, Fondazione IRCCS Istituto Neurologico Carlo Besta, Milan, Italy

**Keywords:** Migraine, Allostatic load, Central sensitization, Complementary treatment, Chronic pain, Environmental factors, Biopsychosocial

## Abstract

Migraine is a complex condition in which genetic predisposition interacts with other biological and environmental factors determining its course. A hyperresponsive brain cortex, peripheral and central alterations in pain processing, and comorbidities play a role from an individual biological standpoint. Besides, dysfunctional psychological mechanisms, social and lifestyle factors may intervene and impact on the clinical phenotype of the disease, promote its transformation from episodic into chronic migraine and may increase migraine-related disability.

Thus, given the multifactorial origin of the condition, the application of a biopsychosocial approach in the management of migraine could favor therapeutic success. While in chronic pain conditions the biopsychosocial approach is already a mainstay of treatment, in migraine the biomedical approach is still dominant. It is instead advisable to carefully consider the individual with migraine as a whole, in order to plan a tailored treatment. In this review, we first reported an analytical and critical discussion of the biological, psychological, and social factors involved in migraine. Then, we addressed the management implications of the application of a biopsychosocial model discussing how the integration between non-pharmacological management and conventional biomedical treatment may provide advantages to migraine care.

## Introduction

**Fig. 1:**
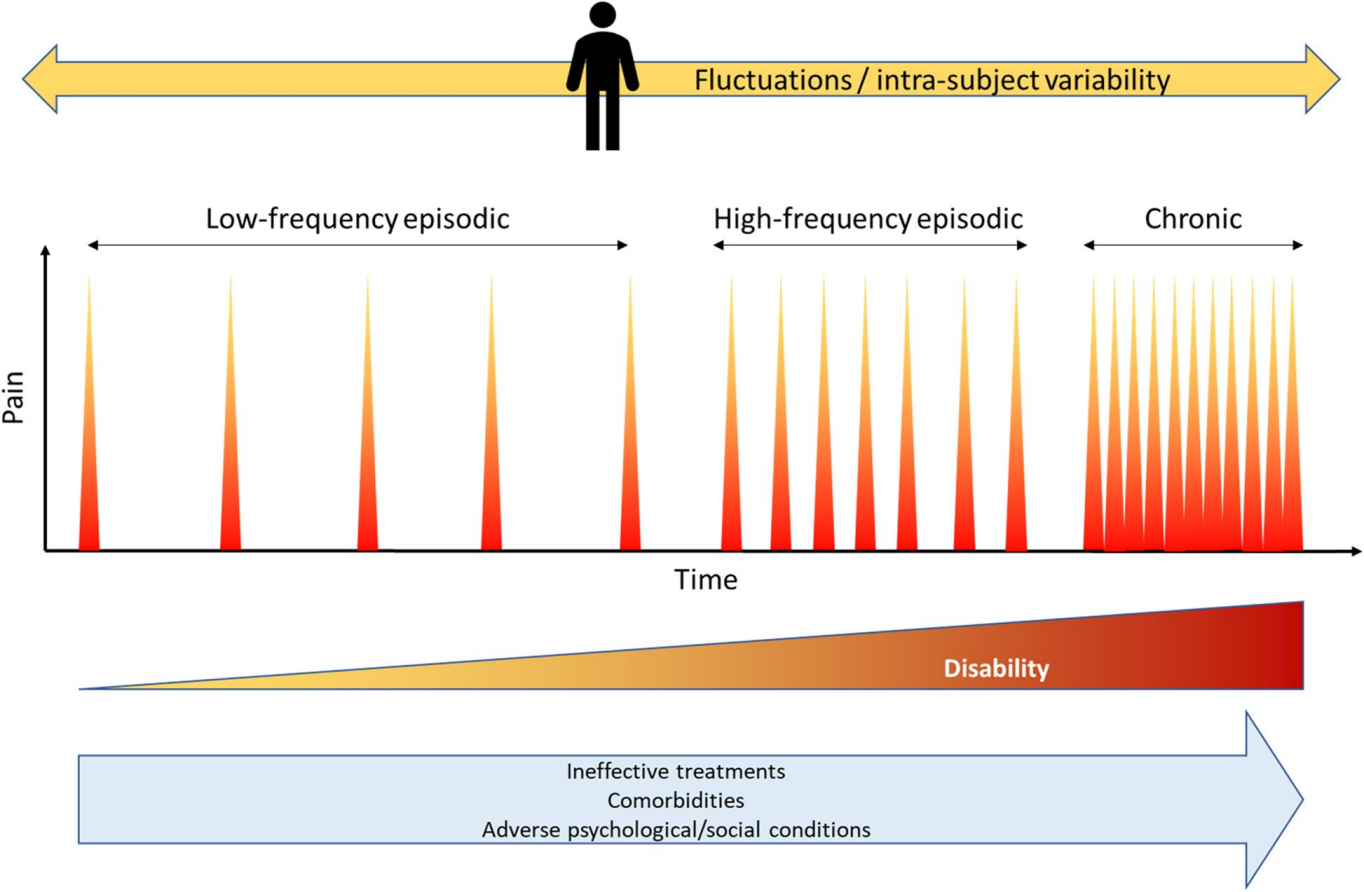
Schematic representation of migraine patterns. Those patterns can change and revert throughout patients’ life

Migraine is a complex disease which may pose substantial burden on individuals. Migraine is the second cause of disability worldwide, and the first cause in young women according to the Global Burden of Diseases (GBD) [1–3]. The recurrent episodes of pain that characterize migraine can have a significant impact on the everyday life of some individuals in terms of lost productivity, family, and social life [4]. Migraine burden is extremely variable across individuals and in the same individual across the life span. The disease ranges from sporadic attacks to daily pain [5]. Between those extremes, individuals may experience all the possible spectrum of attack severity and frequency (Fig. 1). When the individual has more than 15 headache days per month of which 8 have migraine features, the condition is named chronic migraine (CM). Some individuals with migraine also have medication overuse (MO) and others develop resistance or refractories to available treatments [6]. The same individual can experience different patterns of migraine across life and relapses and remissions from and to CM are acknowledged [7]. For many individuals with migraine old age is associated with a resolution of the disease, but for others the disease persists into old age and can have a still disabling pattern [8].

**Fig. 2:**
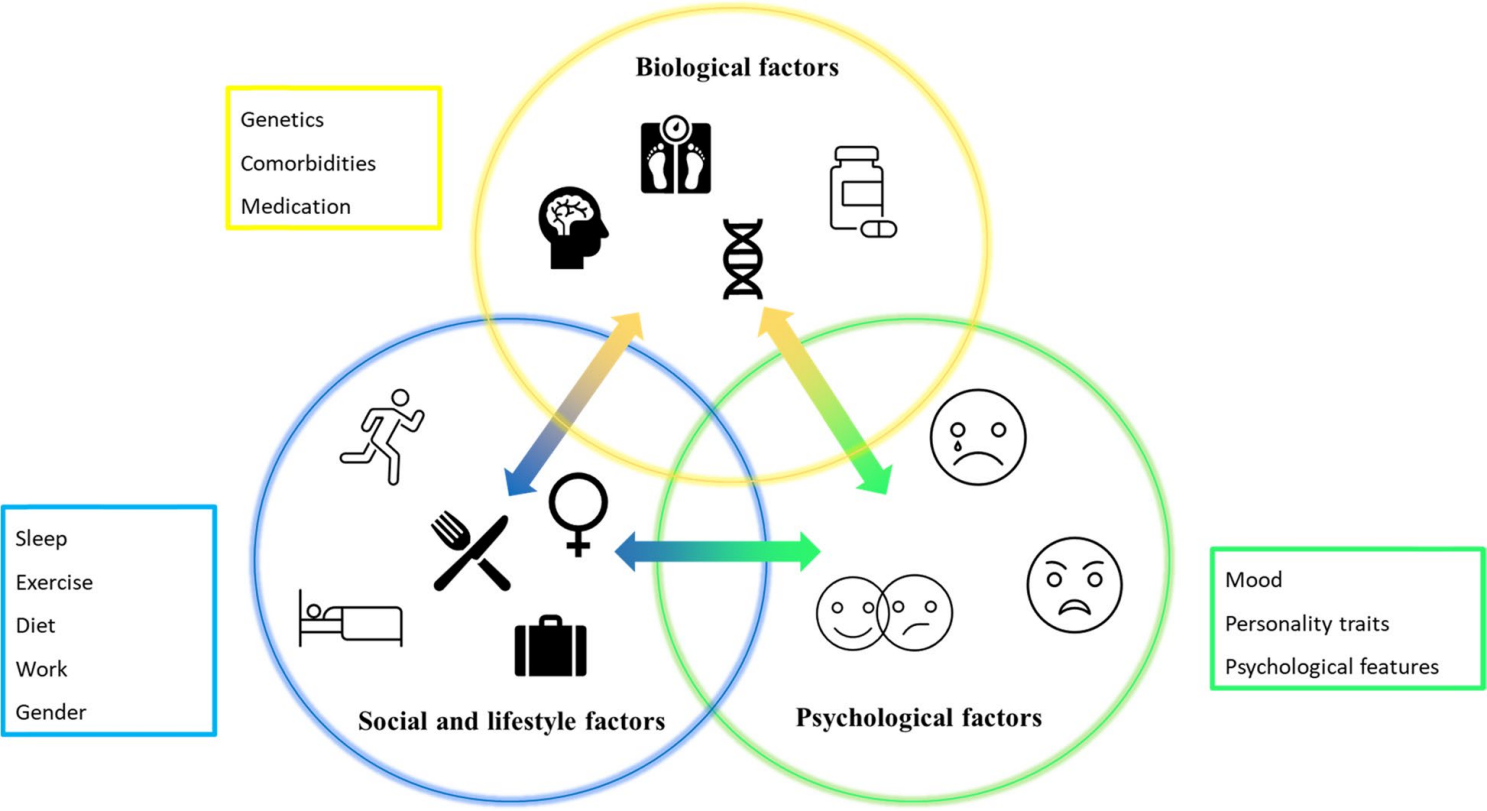
Biological, social, and psychological factors influencing migraine

Migraine arises out of the interaction between biological mechanisms, i.e., genetic loci association, and social, lifestyle and psychological factors (Fig. 2). In fact, on one side genetic predisposition, as demonstrated by migraine typical familial aggregation [8], together with the co-existence of some comorbid conditions are important in the disease [9–12]. On the other side, it cannot be ignored that migraine course also depends on additional complex factors that move far beyond biology [12, 13]. In the present review, we will first summarize the biological and psychosocial factors that interact in determining migraine pattern and individual burden. We will then discuss how a comprehensive approach to the disease, the biopsychosocial (BPS) approach, needs to be better studied and applied to improve the care of individuals with migraine and where possible prevent CM, MO, and drug resistance.

## The BPS model and its application in chronic pain

The BPS is embedded in the definition of health of the World Health Organization of 1948 where Health is a state of complete physical, mental and social well-being and not merely the absence of disease or infirmity. The BPS model was then detailed by the World Health Organization with the International Classification of Functioning Disability and Health (ICF) [9], which addresses the complex interaction between health conditions, individuals and the environment in which individuals conduct their lives, to understand health outcomes in terms of disability. This model tries to overcome the biomedical approach, centered on purely biological mechanisms, by introducing a bottom-up approach which from the biological mechanism considers the individual’s psychological factors and social influences. The model was initially proposed for psychiatric disorders and then applied to pain conditions [10, 11].

In the BPS approach, the “treatment” does not only include pharmacological interventions, but also a personalized and comprehensive assessment and management of factors that may influence the outcome of the disorder. For chronic diseases, diagnosis can remain the same along the whole life while functioning and disability can be modified by acting on the person or the hindering environmental factors.

While the BPS model is highly regarded as an important approach to chronic pain syndromes, it is much less applied and studied in the field of migraine. As a chronic disorder with episodic attacks of intense pain and unpleasant associated symptoms, migraine can provoke a disruption of any aspect of individuals’ life [12–14] The lack of a systematic application of the BPS model to migraine is surprising also considering the similarities between migraine and other pain conditions. High frequency migraine, and especially CM, have some features in common with *nociplastic pain*, which consists in augmented sensory processing and reduced activation of inhibitory pathways. This results in an increased vulnerability of the brain to non-painful sensory stimuli [15]. The main mechanism of both CM and nociplastic pain is c*entral sensitization* [16–18], defined as the perception of pain outside the peripheral tissue where the pain initiates [19].

Notably, individuals with chronic pain conditions such as fibromyalgia, temporomandibular joint disorders, or headaches, have high chances of presenting symptoms of several pain conditions together [20]. The comorbidity between migraine and syndromes characterized by central sensitization, such as fibromyalgia, is frequent [21], further supporting the presence of shared pathophysiology between the disorders.

## Biological mechanisms underlying migraine

The complex interactions between the individual and the environment that are stressed by the BPS approach well fit with the pathophysiology of migraine. Migraine mechanisms involve many areas of the central and peripheral nervous system, and an alteration in brain circuit function which is dynamic [22]. Rather than a dysfunction in a single area of the brain, migraine can be regarded as a dysfunction of sensory processing which ultimately generates episodes of pain [23]. Functional neuroimaging showed that migraine is related to a heightened connectivity among the different sensory areas of the brain [24]. Thus, migraine is regarded as a “connectopathy” rather than a disease arising from dysfunctioning of specific areas of the brain. Interestingly, the same technique also showed a heightened connection between sensory areas and areas regulating affective processes including the limbic system [25–28], which is implied in pain processing and in the regulation of emotional life. This may explain migraineurs’ susceptibility to external triggers causing sensory overload; those triggers may modify brain circuits functioning [29–31]. Individuals with migraine show a decreased threshold for several sensory stimuli, including sensory, pain, thermal, visual, auditory, and olfactory ones, which well correlates with the symptoms of increased sensitivity to light, noise, and odors reported by migraineurs not only during, but also between their attacks. Reduced habituation to sensory stimuli increases the susceptibility of migraineurs to *allostatic load* [32] i.e., a reduced ability to adapt to prolonged and/or repeated stressors [33]. In the case of migraine, headache episodes themselves might act as repeated stressors. If frequency of migraine episodes is particularly high, it might ultimately impair the ability of the brain to adapt to other stressors, including external stimuli [33, 34].

An important mechanism of migraine is peripheral sensitization of the trigeminovascular system, a series of structures including several sensory afferents from the cranium and meninges [35, 36]. Peripheral sensitization implies nociceptive activation in several structures located outside the brain, including the extracranial and pial vessels and the meninges [37]. A widely studied mediator of peripheral sensitization in the trigeminovascular system is calcitonin gene-related peptide (CGRP), which can induce and maintain sterile inflammation in the trigeminovascular system [36, 38, 39]. The peripheral action of CGRP is the target of the recently developed migraine-specific preventive treatments, monoclonal antibodies and gepants [40, 41].

Notably, there is a relationship between peripheral and central sensitization in migraine. The repeated exposure to noxious stimuli at the periphery can activate second-order trigeminal neurons [39, 42–44]. This activation might lead in the long term to a lower threshold for pain perception at the level of the brain, which is the basis for central sensitization. In migraine, the most identifiable clinical marker of central sensitization is cutaneous allodynia, which is the perception of pain in response to non-noxious stimulation of the skin [45]. Cutaneous allodynia can be favored by stressful events, as suggested by both animal [46] and human studies [47], and it is associated with the transition from episodic migraine to CM [48].

## Biological mechanisms beyond migraine: comorbidities

**Fig. 3:**
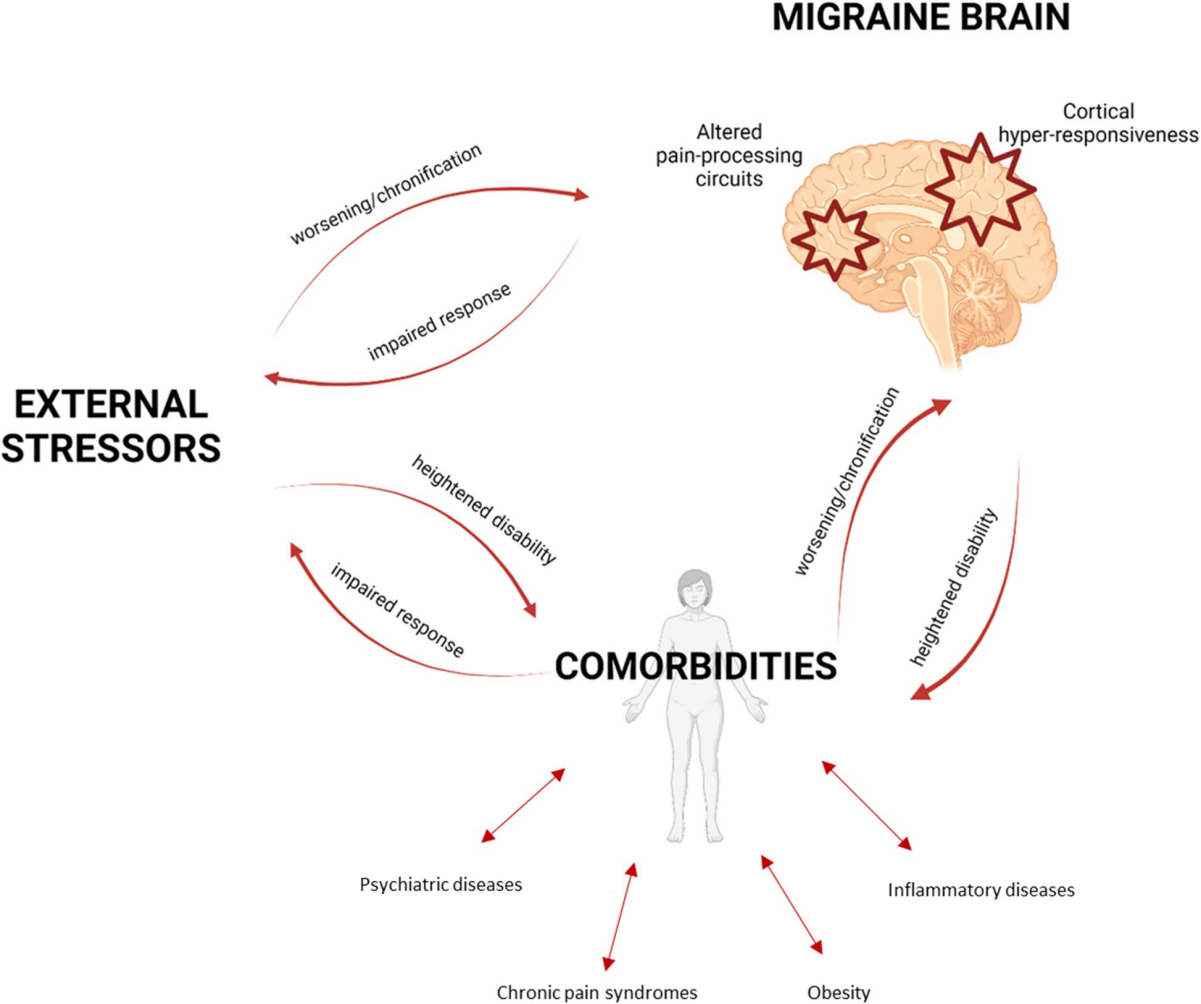
The vicious cycle of migraine comorbidities and stressors. Created with https://biorender.com/

A higher-than-expected frequency of several diseases has been reported in migraine, often with a bidirectional association [49–56] (Fig. 3). This association is attributable to two explanations: 1) migraine shares a common biological mechanism with some other diseases and for this reason they coexist in some individuals; 2) the comorbidity can have an adverse impact on migraine and, thus, migraine-predisposed individuals with the comorbidity have an overt manifestation of migraine or high migraine burden, thus facilitating migraine recognition. A further potential—but less likely—mechanism is that migraine facilitates other diseases. Regarding the first postulated explanation, we can provide the example of psychiatric comorbidities, epilepsy, or sleep disturbances [57–59]. Regarding the second postulated explanation, we can include obesity and inflammatory diseases [56, 60–62]. Irrespectively of the presence or not of shared mechanisms, it is a fact that several treatments which are commonly used for migraine prophylaxis have been repurposed from other diseases. This brings an advantage for patients, namely the possibility to rely on treatments which can positively impact on both migraine and its comorbidities, thus reducing the burden associated to both diseases [49].

### Comorbidities sharing a common biology with migraine

Psychiatric comorbidities associated with migraine include anxiety, depression, post-traumatic stress disorder, substance use disorder, bipolar disease, and even psychosis [55, 63]. A recent meta-analysis [49], based on 4.19 million participants of whom 3.59 (i.e. 86%) with migraine as primary headache diagnosis, showed that the pooled prevalence of depression was 23% (95% CI: 20–26%), of anxiety was 25% (95% CI: 22–28%), both being six-fold the rates observed in the estimates of the 2019 wave of the GBD study [3]. The association with psychiatric disorders is even higher in individuals with MO [64] and in those with migraine with aura [65]. The common underlying circuits that may be relevant to the comorbidity between psychiatric disorders and migraine include the serotonergic pathways, which regulate both mood and pain, and dopaminergic pathways which are implied in the regulation of behavior. Additionally, the autonomic nervous system and the hypothalamus-pituitary axis (HPA) might play a role in those comorbidities [55, 63]; the hypothalamus regulates vegetative functions and is supposedly a migraine generator. Functional magnetic resonance imaging data show that brain areas that can regulate both mood and pain, such as the medial prefrontal cortex, present a similar connectivity in individuals with depression and migraine [66]. There is an association, which can be bidirectional, between high-frequency migraine and depression or anxiety [67–72]. It has been also shown that psychiatric conditions, and mostly depression, favor migraine chronification [73] and may predict poor response to migraine preventive treatments such as onabotulinumtoxinA [74]. Besides, having common underlying mechanisms, migraine and psychiatric disturbances can interact and lead to an adverse phenotype of one another with a mechanism of circular causality (Fig. 3). The phenotypical similarities and shared biological traits between psychiatric comorbidities and pain syndromes are relevant on the treatment perspective. Individuals with multiple pain syndromes (e.g., chronic fatigue syndrome, fibromyalgia, and temporomandibular disorder) present symptoms in comorbidities such as sleep disorders, depression and anxiety as well as those with migraine [75–77].

### Comorbidities impacting on migraine

Obesity is a useful paradigm to understand the role of comorbidities that may have an impact on the course of migraine. A large body of literature shows that obesity is highly prevalent among individuals with the most severe forms of migraine. Obesity itself is a factor associated with migraine chronification [61, 78, 79]. Several mechanisms may explain the association between obesity and CM, including the release of neurotransmitters and proinflammatory adipokines from fat tissue and insulin resistance. Hence, reverting obesity when present may represent an adjunct management strategy for migraine. Studies have demonstrated that weight loss in obese individuals has been associated with migraine improvement [80–82].

Chronic inflammatory rheumatic diseases (e.g., rheumatoid arthritis, psoriatic arthritis, and spondyloarthritis), fibromyalgia and low back pain may also occur in comorbidity with migraine [54, 55]. As shown by the results of a recent meta-analysis [49], the pooled prevalence of arthritis, fibromyalgia and back pain was, respectively, 12% (95% CI: 9–16%), 26% (95% CI: 8–50%), and 46% (95% CI: 20–72%). Rheumatologic diseases are characterized by the presence of systemic inflammation, which could potentiate the neurogenic inflammation of migraine mediated by peripheral CGRP release, and mast cell degranulation [56]. Additionally, many rheumatologic diseases are associated with pain, which could contribute to central sensitization and further worsening of migraine [83, 84].

## Psychological factors

Individuals with migraine can have peculiar psychological and cognitive patterns. Psychological factors can contribute to migraine onset, chronification, development of MO, and response to treatment. Additionally, the presence of certain psychological features increases the level of migraine-related disability, severity of symptoms, and perceived burden of the disease.

### Psychological features of individuals with migraine

Migraineurs seemingly have a unique personality profile that influences – and is influenced by – the perception of their recurrent pain. Certain personality traits could influence the onset of the migraine attack or promote the progression of the disease. According to psychobiological model, migraineurs have high level of harm avoidance and persistence, and a lower level of self-directedness compared to non-migraineurs [85]. Harm avoidance is characterized by behavioural inhibition, excessive worry, pessimism, and introversion. A meta-analysis on personality traits showed that migraine sufferers have a greater apprehension of future problems, avoidance behaviours, and rapid fatigue if compared with non-migraineurs [86]. Migraineurs also tend to have persistence traits, which characterize ambitious, determined individuals who persevere despite frustration or fatigue; they are also characterized by the tendency to maintain unrewarded behaviours, high rigidity, and obsessiveness [85]. Notably, migraine sufferers with persistence traits have poor coping skills and are vulnerable to stress. According to the Eysenck’s Three Factors Models [87], neuroticism is a personality trait that represents the tendency to instability, and feelings such as worry, fear, anger and frustration. Several studies report a higher presence of neuroticism in migraineurs than in non-migraineurs, and lower levels of extraversion [67, 88, 89]. Neuroticism is related to the tendency to experience negative emotions and could moderate the severity of depressive symptomatology and leads to anxiety symptoms. Pain catastrophizing is a cognitive pattern frequently enacted by individuals with migraine [90] and is often the target of cognitive-behavioural therapies (CBT). It consists in the belief that the pain is completely uncontrollable and worse than experienced, exacerbating the feeling of helplessness in response to pain. Pain catastrophizing in migraineurs appears to be a predictor of impaired functioning and quality of life, independent of the presence of other psychological variables (e.g., anxiety and depression) [90].

### Psychological factors associated with migraine chronification

The GRIM2005 study described the psychological variables associated with CM. In individuals with CM, compared with those with episodic migraine, the perceived impact of headache is greater; they exhibit higher levels of emotional distress, worse coping strategies, and a more externalized locus of control. CM is associated with increased catastrophizing of pain, leading to the implementation of maladaptive coping strategies, such as increased support seeking and avoidance strategies. This leads to an individual's inability to manage his or her own pain, relying on others to try to control the pain symptoms [91]. An externalized locus of control is found in CM; it reflects the feeling that the onset and course of migraine attacks is uncontrollable. Individuals with CM find it more difficult to identify the factors that trigger attacks than individuals with episodic migraine, thus increasing the idea that their migraine is controlled by external or random factors. A fear-avoidance model has been extensively studied in chronic pain conditions. In this model, chronic avoidance behavior, reciprocal avoidance, characterized by increased attention to potentially harmful stimuli, and shared vulnerability, characterized by increased sensitivity to anxiety leading to increased attention to pain, are enacted [92]. Fear and avoidance of pain increases disability and promotes progression from acute to chronic pain [93, 94]. Regarding primary headaches, a greater presence of fear of pain was found in CM compared with episodic migraine [94]. Fear of pain appears to be a predictor of headache-related disability even after controlling for variables such as pain, emotional distress, self-efficacy, and locus of control [94].

### Psychological factors, medication overuse, and response to migraine treatments

Certain dysfunctional cognitive patterns, such as pain catastrophizing or anticipatory anxiety could lead to deficit in controlling substance intake, resulting in compulsive drug-taking. Applied to migraine, this can lead to overuse of symptomatic drugs such as analgesics and triptans and increase the likelihood of developing MO.Presence of psychiatric disorders was reported for 68% of individuals with MO; in particular, migraineurs with cluster B personality disorders (e.g. borderline or narcissistic personality disorders) may enact behaviours on pain control and treatment control, resulting in MO [95]. In addition, having been exposed to stressful events in childhood (emotional traumas) has been shown to have a negative impact on the outcome of detoxification therapy in individuals with MO [96].

Psychological factors may influence the response to drug treatment, increasing the likelihood of developing refractory migraine. The presence of personality disorders belonging to Cluster C (e.g. obsessive–compulsive or dependent personality disorders), along with anxiety disorder, stressful events, and alexithymic traits seem to be determinants of erenumab treatment failure in individuals with CM [97].

Finally, some traits seem to be specific to a peculiar group of chronic migraineurs, i.e., those who experience frequent relapses into CM and MO. In addition to the aforementioned depressive symptoms, which also predicted relapse into CM [98] lack of awareness on the severity of their problem, the perception of lack of control on their health status, the lack of hope on possibility to improve, a passive approach towards coping strategies and, finally, being forced to high-functioning in daily life were also found in a qualitative analysis [99].

## Socio-demographic and lifestyle factors

The biology of migraine itself predisposes to the development of a heightened interaction between the individual and the environment, an interaction which could become dysfunctional.

A high migraine burden is associated with specific social and lifestyle factors. Many factors related to lifestyle such as sleep, exercise, and diet are modifiable and may contribute to an effective management of migraine if adequately corrected.

### Socioeconomic status

Low socioeconomic status showed an association not only with a high occurrence, but also with a high frequency of migraine. Low socioeconomic status appears to be bidirectionally associated with migraine. A low income causes greater difficulty in accessing medical care and increased stress, which, as mentioned earlier, increases the likelihood of developing the disease or influences its progression. On the other hand, a disabling disease such as migraine can cause a decline in social status because an individual may have problems in school or work performance [100, 101].

### Working environment and habits

Migraine affects individuals more during working age, so it is important to evaluate work-related characteristics that may worsen the condition. Highly stressful jobs can influence the migraine condition [102]. Shift workers are the category that is most prone to developing migraine, due to dysregulation of circadian rhythms. Migraine has a higher prevalence among those who work night shifts [103, 104]. Similarly, the work environment can trigger or worsen migraine. Possible triggers in the workplace are lights, the brightness of the computer screen or noise [105]. Additionally, in individuals with CM, prolonged computer use during work may aggravate the condition [106].

It has been observed that jobs in health care (e.g. nurses) that involve a higher stress load increase the likelihood of developing migraine [107].

The impact of migraine on employment ca ben measured in terms of absenteeism (i.e. lost workdays) and presenteeism (i.e. days worked with reduced ability) and it is relevant also in connection to the main driver of migraine social cost, i.e. indirect cost, which accounted for 93% of cost in episodic migraine [108], but much less, namely 51.5%, among patients with CM associated to MO at the time point of withdrawal [109]. Presenteeism is surely much more or relevance and, in order to be measured, specific questionnaires exist, such as the Migraine Work and Productivity Loss Questionnaire [110], the Work Productivity and Activity Impairment [111], and the HEADWORK [112].

### Sex and gender

Sex and gender differences in migraine are well-known. Women are up to three times more likely to develop migraine compared to men [113]. Gender can be considered both a biological and a social factor. In fact, the association between migraine and female gender is strongly mediated by the action of hormones; the disorder usually appears after menarche, increases its burden before menstruation, while symptoms tend to improve after menopause [114]. On the other hand, men with migraine tend to use health care resources less frequently than women [115]. Men and women have different social role expectations, coping abilities, and affective variables which can contribute to the generally higher impact of headache, and mostly migraine, on women than on men [116].

### Sleep disorders

As shown by the Chronic Migraine Epidemiology and Outcomes (CaMEO) study, poor sleep and sleep disturbances are risk factors for transformation from episodic to CM [117]. Several sleep disorders, including insomnia, obstructive sleep apnea syndrome, and restless legs syndrome, can have a bidirectional association with migraine, whereas migraine attacks can disrupt night sleep, while sleep disorders may worsen migraine [59]: such disorders are extremely common, as shown in the aforementioned recent meta-analysis, in fact 48% (95% CI: 42–54%) of patients with primary headaches experienced sleeps disorders [49]. Bad sleep was also associated to presence of migraine headaches among adolescents [118–121].

### Exercise

Living a sedentary life with low levels of exercise is associated with a higher prevalence of migraine [122]. In contrast, regular physical activity can be an effective prophylaxis for migraine [123], probably because of the release of endogenous pain relievers after exercise [122, 124, 125] Physical activity may also promote reversion from CM to episodic migraine [126]. On the other hand, strenuous physical activity can act as a trigger for migraine attacks [124]. To integrate physical activity in the management of migraine, it is best to give attention to warm-up and avoid high intensities, so to avoid the excess of anaerobic metabolism which is the main trigger of migraine [122].

### Diet

As well as for exercise, diet can play a role in migraine. Some foods might trigger migraine [127], even if the trigger action of some foods could be a misinterpretation of migraine prodromes [128, 129]. More important is the role of diet as a whole in individuals with migraine, given the relevance of glucose metabolism in migraineurs’ brains [130, 131]. Diets shifting the brain metabolism to products different from glucose, such as the ketogenic diet, have shown to provide some benefits in migraine management [132, 133] decrease of the hypermetabolic feature of migraineurs’ brain. However, even dietary measures that are less strict than ketogenic diet, including weight loss in obese individuals, low-calorie diets, or fatty acid supplementation, might be able to decrease the burden of migraine [134]. Irregularity with meals consumption was also associated to presence of migraine headaches among adolescents [121].

### External stressors

Studies on chronic musculoskeletal pain have shown that emotional distress and psychosocial stress increase the likelihood of transforming acute pain into chronic pain or influencing long-term outcomes [135]. The presence of prior psychological trauma is associated with a 2.7-fold increased risk in the development of chronic widespread pain [136, 137], as well as in the onset of migraine and in its chronification. In fact, a succession of stressful events at an early age (e.g. abuse, emotional trauma) lead to the onset of migraine in adulthood Higher migraine frequency is associated with higher levels of perceived stress, and 70–80% of migraine suffers report that stress promotes the onset of migraine attack [136–140], as well as in the onset of migraine and in its chronification. In fact, a succession of stressful events at an early age (e.g. abuse, emotional trauma,) lead to the onset of migraine in adulthood [138]. The exact causal relationship linking migraine and stress is not entirely clear. On a pathophysiological point of view, hyperactivation of the sympathetic nervous system and hypothalamic axis, the two systems related to response to stress, could potentially promote migraine attack or affect migraine in other ways [141].

## Migraine management according to the BPS model

A successful migraine strategy to treat migraine should be tailored to the needs of single individuals and consider not only pharmacological management but also management of any other factor which may be relevant to the disease in the individual.

### A multicomponent and multi-layered model

When a diagnosis of migraine is established, treatment has to be targeted to individual needs [142]. Drugs to relieve pain are to be used in all individuals experiencing migraine attacks. The need to establish a pharmacological prevention should be individualized [143] depending on the frequency of migraine attacks and on the disability and impairment in function.

Currently, migraine treatment is mostly focused on pharmacological interventions. However, given that migraine is a multifactorial disease (Fig. 2), it is of the utmost importance to adopt a comprehensive approach to tackle, in everyone, all the factors which are important in determining the impact of the disease and to prevent an adverse course over time.

Inclusive consideration of the individual and different levels of prevention are the main features of the model that derives from the BPS approach. In fact, adopting this approach implies, first, to perform an assessment in all functional domains of individuals’ life (i.e., biological, social, and psychological-behavioural) to assess migraine predisposing, precipitating, perpetuating, and protective factors. Ideally, environmental issues should be taken into consideration as well.

The clinical interview assumes considerable importance in this context. It should be clearly patient-centered and sufficient space should be given to the free expression of the individual's feelings, doubts about treatment and thoughts [144]. Quality of life, and not only pain, is a central theme of the migraine experience, and the clinical interview should focus on this aspect [145]. Improving communication skills of headache clinicians could lead to improved management of individuals with migraine, as well as a reduction in the stigma that characterizes this condition [146–149].

**Fig. 4:**
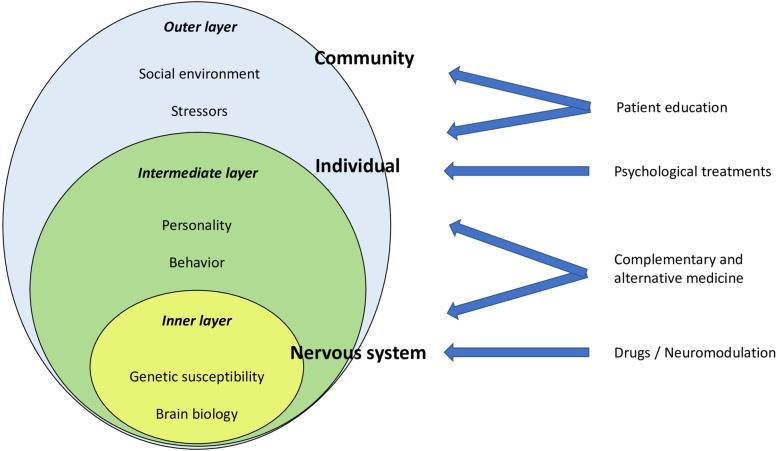
A hierarchical view of the biopsychosocial model as applicable to migraine

Based on the above, a BPS approach to the individual should be multi-layered. At the inner layer, there is the biological constitution of individuals (including comorbidities) and of their brain circuits; at the intermediate layer, there is the psychological and behavioural component of individuals, which directly influence the difficulties they experience in daily chores; finally, the external layer includes the complex interactions between individuals and their social environment, which might further on increase the difficulties in daily life activities (e.g. noisy physical environment which might precipitate migraine headaches, stigma, prejudice), or might on the contrary reduce them (e.g. availability of adequate prophylaxis, flexible timetable in the workplaces, presence of people available to provide support, etc.). The three hierarchical components of the model are in an ever-changing balance between each other (Fig. 4). The biological level is where pharmacological treatments act, the results of such action being however mainly manifest in the intermediate layer. If the psychosocial environment and behaviour of individuals are functional, and pharmacological prevention is effective, the individual is well-managed. If there are psychological-behavioural and/or social dysfunctional factors, the effect of pharmacological treatments is overwhelmed by external triggers, stressors, and comorbidities.

### Inner layer: pharmacological management strategies and treatment of comorbidities

Pharmacological migraine treatment currently includes preventive medical therapies and acute treatments [150, 151]. Among the first group, antidepressants, antiepileptics, calcium-channel blockers, beta-blockers, drugs acting on the CGRP pathway, angiotensin-converting enzyme inhibitor or angiotensin II receptor blocker, nerve blocks, and, for CM, onabotulinumbotxinA are available. Acute pharmacological treatments include ergot alkaloids, triptans, combined medications, non-steroidal anti-inflammatory drugs (NSAIDs), gepants, ditans, and antiemetics or prokinetics [150, 151]. The choice of the most appropriate medication suitable for each person is highly individualized and requires careful consideration based on individuals’ clinical features, concomitant comorbidities, and preferences [142, 152, 153]: however, even with the appropriate use of currently available treatments, individuals may still present unmet therapeutic needs.

Migraine comorbidities should be kept in mind during interview with individuals and should be properly assessed and managed in parallel with pharmacological strategies [49]. Given the complex interactions between migraine and its comorbidities, treating those conditions may result in a better control of migraine [154]. For example, treating obstructive sleep apnea syndrome in migraineurs can improve their headaches by improving sleep quality [155], while weight loss in general can improve migraine [156–158].

### Intermediate layer: education and non-pharmacological approaches

Individual information and education and personal support are mainstays of migraine management which should be always applied to every individual with migraine. Individuals with migraine may face difficulties in understanding the primary nature of the pain and may spend time and resources in performing visits and exams to find a structural cause of the disease. Negative findings from those exams and conflicting opinions from health-care providers who are not expert in headache may be a source of frustration and may lead to adverse health outcomes. Consequently, basic information to explain the nature of the pain and the course of the disease should be provided to all individuals with new diagnosed migraine.

Individuals with migraine may also struggle in accepting and coping with pain, leading to high levels of self-perceived headache-related disability [159] and pain catastrophizing [160]. The variable frequency of symptoms and the largely unpredictable timing of severe migraine attacks can result in individuals feeling that they have no control over their illness [18]. Therefore, individuals should also receive specific coping strategies for migraine management mainly dealing with migraine pattern and trigger monitoring (i.e., diaries), and painkiller use.

In addition to coping and pain management strategies, education on key lifestyle factors is also very helpful and important in reducing migraine. [161]. Basic information for individuals includes avoiding weight gain, or high level of stress, while keeping regular wake sleep cycle and physical exercise.

Non-pharmacological approaches include a variety of treatments [146] in particular: non-invasive neurostimulation techniques [162–165], e.g. Transcranial Magnetic Stimulation (TMS), Supraorbital Nerve Stimulation (SNS), and Transcranial Direct Current Stimulation (tDCS); behavioral approaches [166–170], e.g. CBT, mindfulness-based approaches, such as mindfulness-based stress reduction (MBRS) therapy, acceptance and commitment therapy (ACT), biofeedback and relaxation techniques; nutraceuticals [171–173], such as Butterbur, Coenzyme Q10, Feverfew, Magnesium, and Riboflavin. Finally, Complementary and Alternative Medicine (CAM), i.e. a series of treatments aimed at integrating or replacing standard medical treatments have also recently been investigated in the literature, including traditional Chinese medicine, massage, yoga, or chiropractic care [174–177], whose utilization is however often not disclosed by patients [178]. However, before using any of these treatments, a doctor-patient discussion on their use is recommended, paying attention to adverse events and possible interactions with other current treatments [174]. Behavioral therapies, aimed at stress management, control overuse of medications, catastrophizing reduction, and enhancement of self-efficacy and internal locus of control, have the potential to booster the effectiveness of pharmacological treatments. It is important to note that non-pharmacological approaches produce quantifiable effects on migraine-related brain circuits and other systems implied in the genesis of migraine [179]. Appropriate dietary regimes and weight loss might decrease the systemic inflammation, secretion of adipokines, and oxidative stress [180] leading to sensitization of the trigeminovascular system [80]. Neuroimaging findings have further demonstrated the impact of behavioural treatments, and especially mindfulness, in producing functional modifications in brain areas involved in the cognitive and affective components of pain, including the dorsolateral medial prefrontal cortex, dorsal anterior insula, and anterior midcingulate cortex [181].

### Outer layer: social factors

Social factors are difficult to modify as they depend on factor external to the person and interacting with him/her. An assessment about cultural, environmental, and socioeconomic aspects of a person with migraine should be considered during assessments. Characteristics of or relevant changes in social support networks are often able to modify migraine patterns and should be addressed by supporting individuals in improving their coping abilities and by educating the environment to avoid stigmatizing behaviors against people with migraine, at family, working, societal levels [182]. Some of those factors can be recognized as stressors that may impair the effectiveness of migraine pharmacological treatments. While it is not always possible to change the social environment of individuals with migraine, a useful management strategy for those individuals is to focus on the management of external stressors.

An interesting type of social intervention for migraine sufferers could be the inclusion in support groups formed by healthcare providers and/or other migraine sufferers. This will give them the opportunity to talk and confront themselves with those who share the same pain condition and difficulties, and to have relationships with others outside of family and friends.

In addition, addressing the relationship between the individual with migraine and the treating clinician is an environmental factor that could be facilitator or barrier into the care pathway of the patient.

### BPS management of resistant, refractory or MO migraine

Migraine that is resistant or refractory to pharmacologic prevention is an important issue in clinical practice, not only among headache specialists but also in primary care [183]. Prevention and treatment of those conditions with a traditional biomedical approach can be unsatisfactory. Holistic interventions acting on both the individual and environment could increase the probability of treating CM, refractory migraine, or MO, as already proposed for nociplastic pain. The opportunities of treating difficult-to-treat primary headaches, and mostly MO, with a multidisciplinary team and structured interventions have already been discussed in previous literature [184, 185]. Many of those individuals have an unfavourable psychosocial profile that leads to a negative impact on response to treatments, together with their pharmacogenetic profile. In those individuals, careful consideration of their life experience could unveil the presence of elements – clinical, psychological, and/or sociocultural – that can negatively affect the course and the management of migraine. These elements, which are frequently accounted in the field of chronic pain syndromes, would deserve a higher consideration in headache medicine. Pharmacological management of migraine should be combined with the assessment of factors that, although not strictly linked to migraine, are linked by circular causation to migraine burden in everyday life, for example the likelihood that patients have to face limitations in their activities due to migraine at home, at work or in their leisure time.

### The BPS approach and placebo effect

Strategies to enhance placebo effect and mitigate nocebo effect may offer additional aid to manage migraine in the context of BPS approach. Placebo refers to biologically inactive substances, and the placebo effect is the therapeutic benefit experienced after taking a placebo. The nocebo effect, on the other hand, can be considered the opposite of the placebo effect and occurs when an individual experiences adverse events after the administration of an inactive substance. Underlying these phenomena are cognitive mechanisms of pain modulation, and the placebo response to a large extent also depends on the psychosocial context [186]. Placebo is a relevant issue in migraine trials as it is very high, leading to relatively small margins of efficacy of anti-migraine drugs. A meta-analysis addressing the proportion contextual effect (PCE), i.e. the ratio between the reduction in monthly migraine days in the placebo and in the experimental group – which in this case were eptinezumab, erenumab, fremanezumab and galcanezumab – showed that 66–68% of the achieved was due to contextual factors, including placebo effect [187]. Such a value is slightly higher than that previously observed for valproate and propranolol (57–58%) [188]. In a randomized controlled trial in pediatric population, the very high efficacy of placebo led a non-significant relative effect of active drugs over placebo even in the presence of positive absolute effects in more than 60% of individuals [189]. The presence of a so evident placebo and nocebo effect in migraine is a clear hallmark that non strictly biological factors may play a very important role in response to treatments. Several factors influence placebo effect in migraine, including the way of administration of drugs, individuals’ expectations, blinding, age, gender, and even geography. Discussion between individuals and physicians can lead to both placebo and nocebo effects depending on the way it is carried forward. In clinical practice, creating a proportion of placebo effect is not a confounder like in randomized clinical trials but it is an added value to any treatment; in fact, it could be a useful strategy to improve individuals’ outcomes and ensure improvements in migraine outcomes. Avoiding nocebo effect can contribute as well to successful treatment. Further studies are needed to address strategies to try to maximize the benefits derived from inducing a placebo effect as well as structured strategies to minimize nocebo effect need to be studied.

## Limitations of the BPS model applied to migraine

The BPS model is well applicable to the field of migraine clinics and research because of its circular causality, which explains how the pathogenesis of migraine and its expression in individuals are a series of vicious circles that should be taken into account for the effective management of individuals, although they are difficult to break. This model has many strengths, but also limitations that will be discussed below.

### Why BPS model is considered anti-scientific (by those that do not know it)?

The model proposed by Engel explains psychological and social characteristics to take into account but does not provide structured system of assessment and quantification. There is not a standardized description of optimal strategies to transfer the model in practice. It is argued how the model is vague, too general, and without precise guidelines on how to implement it in clinical practice, and which area is to prioritize. Although it is quite clear that assessments and interventions performed through the BPS model should be conducted by a multidisciplinary team, the ideal skill-mix to obtain an effective and efficient team caring of all the spectrum of individuals with migraine is not yet established. It is also pointed out that physicians often lack training in how to treat the individual from a psychological and social perspective as well [190–192]. Over the years, the BPS model has also been criticized as lacking strong scientific validity also due to the impossibility of planning double-blind trials for psychosocial interventions. Due to its vagueness, the model can also be misused and misinterpreted, with the introduction of reductionist and fragmented approaches that underestimate its intrinsically humanistic value [193]. It has to be taken also into account that the BPS model is a theoretical model: the way in which different parts of it are operationalised can on the contrary be addressed with RCT, either open-label or single-blinded ones, pragmatic trials or real-life studies. Each of these studies, which might deal for example with the evaluation of service organization [194–196], can provide an evaluation of specific parts of the BPS model, relying on specific outcomes. However, to date these limitations of the BPS model can be partially resolved by the introduction of the ICF classification by the World health Organization, which has provided a robust methodology to identify all the elements present in the BPS model and to define precise treatment for each of the components that can be identified as target for specific actions: at body, person, environmental levels.

### Barriers to the implementation of the BPS model in headache care

The full application of a BPS model in individuals with migraine has several barriers.

The first barrier is in the incomplete knowledge of migraine pathophysiology. Despite many decades of research efforts, we still do not have a unified model of migraine pathogenesis like we have in other pain syndromes. This lack of knowledge led to the widespread use of pharmacological treatments that are largely empirical, with the consequence of partial effectiveness and unsatisfactory results. The unmet needs of non-specific pharmacological treatments are coupled with uncertainties regarding the optimal management of non-pharmacological treatments. It is very difficult to obtain high-quality evidence on those treatments as they are usually individualized and rely on individuals’ compliance. For example, we know that behavioural treatments are effective for migraine: however, we should also consider that the variability in schedules, modalities, and individual compliance to behavioural treatments is much higher than that of pharmacological treatments, and likely different in quality. Therefore, we cannot make, now, any recommendation on which is the best behavioural strategy to treat migraine, but we can identify issues that different kind of treatments might approach with benefit for single patients.

A second barrier to the implementation of the BPS model to migraine is in healthcare resource use. Migraine is a very prevalent condition; all individuals with migraine have the right of a correct diagnosis and the best available treatment: however, not all persons with migraine can be treated with an adequate use of health resources, and many have very mild forms of the disease, probably because of a relatively favourable biology and good interaction with psychosocial environment.

A thorough assessment of psychosocial factors should be “mandatory” for those individuals with refractory migraine and those with a substantial impact of migraine on their lives, i.e., for those cases in which the pharmacological approach alone is not enough. Adequate levels of care need to be made available to the largest possible amount of people, whatever this might mean along the continuum of available treatments: therefore, structured headache services are a priority for health systems organization [153, 195, 196]. Environments with no health care for people with migraine, for example could be the worst scenario to improve migraineurs’ life. What is probably the most important barrier to the implementation of the BPS model is the difficulty to effectively change environment putting barriers at level of policies, health care organization, health care provision.

The third barrier is the subjectivity implicit in the BPS model. Ideally, each individual should receive a tailored management plan based on their specific environment. This approach is extremely valid on a clinical point of view, while it could be uneasy to put in place and monitor by the means of research which aim at standardizing results and procedures. The health status of a single individual is the result of many influencing variables; in a research context, the analysis of huge datasets and the deployment of novel methods such as artificial intelligence could lead to considering all possible health and health-related factors and to personalized treatment strategies. One of the future challenges of clinical research in the field of headache will be to provide general recommendations for an inclusive and individualized management of individuals with migraine while maintaining consensus on standard procedures.

## The relationship between the BPS model and personalized medicine

At a first glance, the BPS model well fits with the demands of the so-called “personalized medicine” which aims to deliver the right intervention, for the right individual, at the right time [197]. Such an approach, if brought to its extreme boundaries, might led to scaling up studies that focus on a single person, i.e. the so-called N-of-1 trials [198]. Recognizing and managing all the factors potentially influencing migraine could allow a more precise phenotyping of migraine and targeted treatments. A similar relationship between the BPS model and precision medicine has been advocated for chronic pain syndromes [10]. It should be noted that the BPS model and precision medicine have different basic assumptions. Inevitably, the BPS model has a subjective component which is based on the individual’s psychological functioning and social environment, while the aim of precision medicine is to “objectify” the very high number of variables that can have an impact on the individual’s health. The potential discrepancies between the BPS model and precision medicine have been reported in the field of chronic pain [193], but are equally applicable to migraine.

## Conclusions

Migraine is caused by a pathological functioning and interactions of brain circuits. This mechanism is driven by a multiplicity of factors deriving from gene predisposition and factors which may occur over life. The expression of migraine is not fixed as it results from the management of those factors. There is a common milieu between migraine, psychiatric comorbidities, and some behavioural and psychological traits, all of which may adversely affect the course of the disease and enhance dysfunctional pain processing. This can lead to self-perpetuating, maintenance and enhancement of a vicious circle of dysfunctional central circuits associated with pain, emotions, and behaviours. Moreover, people with migraine might have hindering environments that could worsen the course of the diseases, such as being exposed to stigma, lacking support from relevant others, or dealing with complex interactions at work. Further studies will make it possible to determine the mechanisms underlying the relationship between the biological mechanisms of individuals with migraine and the interaction with the psychosocial environment.

Deep investigation of the complex milieu of neuroinflammation and related connectivity changes may unveil an inter-individual variability in signaling pathways that goes beyond clinical differences and could provide targets for mechanism-based precision medicine approaches.

Although many lines of research and clinical practice clearly suggest the potentialities of the BPS model applied to migraine, applying the model is complex as it implies an inclusive assessment of individuals, their needs, and their life as well as of all the environmental elements that could be modified. In our opinion, striving towards better understanding of this interaction is a research and clinical priority in the field of migraine.

## Data Availability

The study does not contain original data.

## Abbreviations

ACT: Acceptance and Commitment Therapy
BPS: Bio-Psycho-Social
CAM: Complementary and Alternative Medicine
CaMEO: Chronic Migraine Epidemiology and Outcomes
CBT: Cognitive-Behavioural Therapy
CGRP: Calcitonin Gene-Related Peptide
CM: Chronic Migraine
GBD: Global Burden of Disease study
HPA: Hypothalamus-Pituitary Axis
MBRS: Mindfulness-Based Stress Reduction therapy
MO: Medication Overuse
NSAIDs: Non-Steroidal Anti-Inflammatory Drugs
PCE: Proportion Contextual Effect
SNS: Supraorbital Nerve Stimulation
tDCS: Transcranial Direct Current Stimulation
TMS: Transcranial Magnetic Stimulation

## References

[1] Safiri S, Pourfathi H, Eagan A, Mansournia MA, Khodayari MT, Sullman MJM (2022). Global, regional, and national burden of migraine in 204 countries and territories, 1990 to 2019. Pain.

[2] Steiner TJ, Stovner LJ, Jensen R, Uluduz D, Katsarava Z (2020). Migraine remains second among the world’s causes of disability, and first among young women: findings from GBD2019. J Headache Pain.

[3] GBD 2019 Diseases and Injuries Collaborators (2020) Global burden of 369 diseases and injuries in 204 countries and territories, 1990–2019: a systematic analysis for the Global Burden of Disease Study 2019. Lancet 396:1204–1222 10.1016/S0140-6736(20)30925-9 PMC7567026 33069326

[4] Leonardi M, Raggi A (2019). A narrative review on the burden of migraine: when the burden is the impact on people’s life. J Headache Pain.

[5] Olesen J (2018). International classification of headache disorders. Lancet Neurol.

[6] Sacco S, Braschinsky M, Ducros A, Lampl C, Little P, van den Brink AM (2020). European headache federation consensus on the definition of resistant and refractory migraine : Developed with the endorsement of the European Migraine & Headache Alliance (EMHA). J Headache Pain.

[7] Serrano D, Lipton RB, Scher AI, Reed ML, Stewart WBF, Adams AM (2017). Fluctuations in episodic and chronic migraine status over the course of 1 year: implications for diagnosis, treatment and clinical trial design. J Headache Pain.

[8] Lipton RB, Bigal ME, Diamond M, Freitag F, Reed ML, Stewart WF (2007). Migraine prevalence, disease burden, and the need for preventive therapy. Neurology.

[9] World Health O (2001). International classification of functioning, disability and health : ICF.

[10] Cohen SP, Vase L, Hooten WM (2021). Chronic pain: an update on burden, best practices, and new advances. Lancet.

[11] Andrasik F, Flor H, Turk DC (2005). An expanded view of psychological aspects in head pain: the biopsychosocial model. Neurol Sci.

[12] Buse DC, Fanning KM, Reed ML, Murray S, Dumas PK, Adams AM (2019). Life with migraine: effects on relationships, career, and finances from the Chronic Migraine Epidemiology and Outcomes (CaMEO) study. Headache.

[13] D’Amico D, Tepper SJ, Guastafierro E, Toppo C, Leonardi M, Grazzi L (2020). Mapping assessments instruments for headache disorders against the ICF biopsychosocial model of health and disability. Int J Environ Res Public Health.

[14] Raggi A, Giovannetti AM, Quintas R, D’Amico D, Cieza A, Sabariego C (2012). A systematic review of the psychosocial difficulties relevant to patients with migraine. J Headache Pain.

[15] Fitzcharles MA, Cohen SP, Clauw DJ, Littlejohn G, Usui C, Hauser W (2021). Nociplastic pain: towards an understanding of prevalent pain conditions. Lancet.

[16] Su M, Yu S (2018). Chronic migraine: a process of dysmodulation and sensitization. Mol Pain.

[17] Mungoven TJ, Henderson LA, Meylakh N (2021). Chronic migraine pathophysiology and treatment: a review of current perspectives. Front Pain Res (Lausanne).

[18] Andreou AP, Edvinsson L (2019). Mechanisms of migraine as a chronic evolutive condition. J Headache Pain.

[19] Nijs J, George SZ, Clauw DJ, Fernández-de-las-Peñas C, Kosek E, Ickmans K (2021). Central sensitisation in chronic pain conditions: latest discoveries and their potential for precision medicine. Lancet Rheumat.

[20] Maixner W, Fillingim RB, Williams DA, Smith SB, Slade GD (2016). Overlapping chronic pain conditions: implications for diagnosis and classification. J Pain.

[21] de Tommaso M, Sardaro M, Serpino C, Costantini F, Vecchio E, Prudenzano MP (2009). Fibromyalgia comorbidity in primary headaches. Cephalalgia.

[22] Charles A (2018). The pathophysiology of migraine: implications for clinical management. Lancet Neurol.

[23] Goadsby PJ, Holland PR (2019). An update: pathophysiology of migraine. Neurol Clin.

[24] Meylakh N, Henderson LA (2022). Exploring alterations in sensory pathways in migraine. J Headache Pain.

[25] Mungoven TJ, Marciszewski KK, Macefield VG, Macey PM, Henderson LA, Meylakh N (2022). Alterations in pain processing circuitries in episodic migraine. J Headache Pain.

[26] Huang X, Zhang D, Wang P, Mao C, Miao Z, Liu C (2021). Altered amygdala effective connectivity in migraine without aura: evidence from resting-state fMRI with Granger causality analysis. J Headache Pain.

[27] Chen Z, Chen X, Liu M, Dong Z, Ma L, Yu S (2017). Altered functional connectivity of amygdala underlying the neuromechanism of migraine pathogenesis. J Headache Pain.

[28] Maizels M, Aurora S, Heinricher M (2012). Beyond neurovascular: migraine as a dysfunctional neurolimbic pain network. Headache.

[29] Silvestro M, Tessitore A, Di Nardo F, Scotto di Clemente F, Trojsi F, Cirillo M (2022). Functional connectivity changes in complex migraine aura: beyond the visual network. Eur J Neurol.

[30] Lee MJ, Park BY, Cho S, Kim ST, Park H, Chung CS (2019). Increased connectivity of pain matrix in chronic migraine: a resting-state functional MRI study. J Headache Pain.

[31] Qin Z, Su J, He XW, Ban S, Zhu Q, Cui Y (2020). Disrupted functional connectivity between sub-regions in the sensorimotor areas and cortex in migraine without aura. J Headache Pain.

[32] McEwen BS, Stellar E (1993). Stress and the individual. Mechanisms leading to disease. Arch Intern Med.

[33] Borsook D, Maleki N, Becerra L, McEwen B (2012). Understanding migraine through the lens of maladaptive stress responses: a model disease of allostatic load. Neuron.

[34] Blumenfeld A, Durham PL, Feoktistov A, Hay DL, Russo AF, Turner I (2021). Hypervigilance, allostatic load, and migraine prevention: antibodies to CGRP or receptor. Neurol Ther.

[35] Noseda R, Burstein R (2013). Migraine pathophysiology: anatomy of the trigeminovascular pathway and associated neurological symptoms, cortical spreading depression, sensitization, and modulation of pain. Pain.

[36] Ashina M, Hansen JM, Do TP, Melo-Carrillo A, Burstein R, Moskowitz MA (2019). Migraine and the trigeminovascular system-40 years and counting. Lancet Neurol.

[37] Olesen J, Burstein R, Ashina M, Tfelt-Hansen P (2009). Origin of pain in migraine: evidence for peripheral sensitisation. Lancet Neurol.

[38] Messlinger K (2018). The big CGRP flood - sources, sinks and signalling sites in the trigeminovascular system. J Headache Pain.

[39] Iyengar S, Johnson KW, Ossipov MH, Aurora SK (2019). CGRP and the trigeminal system in migraine. Headache.

[40] Capi M, De Angelis V, De Bernardini D, De Luca O, Cipolla F, Lionetto L (2021). CGRP receptor antagonists and 5-HT1F receptor agonist in the treatment of migraine. J Clin Med.

[41] Sacco S, Amin FM, Ashina M, Bendtsen L, Deligianni CI, Gil-Gouveia R (2022). European Headache Federation guideline on the use of monoclonal antibodies targeting the calcitonin gene related peptide pathway for migraine prevention – 2022 update. J Headache Pain.

[42] Bernstein C, Burstein R (2012). Sensitization of the trigeminovascular pathway: perspective and implications to migraine pathophysiology. J Clin Neurol.

[43] Burstein R, Noseda R, Borsook D (2015). Migraine: multiple processes, complex pathophysiology. J Neurosci.

[44] Iyengar S, Ossipov MH, Johnson KW (2017). The role of calcitonin gene-related peptide in peripheral and central pain mechanisms including migraine. Pain.

[45] Louter MA, Bosker JE, van Oosterhout WP, van Zwet EW, Zitman FG, Ferrari MD (2013). Cutaneous allodynia as a predictor of migraine chronification. Brain.

[46] Hawkins JL, Moore NJ, Miley D, Durham PL (2018). Secondary traumatic stress increases expression of proteins implicated in peripheral and central sensitization of trigeminal neurons. Brain Res.

[47] You DS, Creech SK, Meagher MW (2016). Enhanced area of secondary hyperalgesia in women with multiple stressful life events: a pilot study. Pain Med.

[48] Bigal ME, Lipton RB (2009). What predicts the change from episodic to chronic migraine?. Curr Opin Neurol.

[49] Caponnetto V, Deodato M, Robotti M, Koutsokera M, Pozzilli V, Galati C (2021). Comorbidities of primary headache disorders: a literature review with meta-analysis. J Headache Pain.

[50] Ng CYH, Tan BYQ, Teo YN, Teo YH, Syn NLX, Leow AST (2022). Myocardial infarction, stroke and cardiovascular mortality among migraine patients: a systematic review and meta-analysis. J Neurol.

[51] Burch RC, Buse DC, Lipton RB (2019). Migraine: epidemiology, burden, and comorbidity. Neurol Clin.

[52] Altamura C, Corbelli I, de Tommaso M, Di Lorenzo C, Di Lorenzo G, Di Renzo A (2021). Pathophysiological bases of comorbidity in migraine. Front Hum Neurosci.

[53] Sacco S, Cerone D, Carolei A (2008). Comorbid neuropathologies in migraine: an update on cerebrovascular and cardiovascular aspects. J Headache Pain.

[54] Sacco S, Olivieri L, Bastianello S, Carolei A (2006). Comorbid neuropathologies in migraine. J Headache Pain.

[55] Minen MT, Begasse De Dhaem O, Kroon Van Diest A, Powers S, Schwedt TJ, Lipton R (2016). Migraine and its psychiatric comorbidities. J Neurol Neurosurg Psychiatry.

[56] Rivera-Mancilla E, Al-Hassany L, Villalón CM, MaassenVanDenBrink A (2021). Metabolic Aspects of migraine: association with obesity and diabetes mellitus. Front Neurol.

[57] Keezer MR, Bauer PR, Ferrari MD, Sander JW (2015). The comorbid relationship between migraine and epilepsy: a systematic review and meta-analysis. Eur J Neurol.

[58] Duko B, Ayalew M, Toma A (2020). The epidemiology of headaches among patients with epilepsy: a systematic review and meta-analysis. J Headache Pain.

[59] Tiseo C, Vacca A, Felbush A, Filimonova T, Gai A, Glazyrina T (2020). Migraine and sleep disorders: a systematic review. J Headache Pain.

[60] Westgate CSJ, Israelsen IME, Jensen RH, Eftekhari S (2021). Understanding the link between obesity and headache- with focus on migraine and idiopathic intracranial hypertension. J Headache Pain.

[61] Peterlin BL, Sacco S, Bernecker C, Scher AI (2016). Adipokines and migraine: a systematic review. Headache.

[62] Moisset X, Giraud P, Dallel R (2021). Migraine in multiple sclerosis and other chronic inflammatory diseases. Rev Neurol.

[63] Dresler T, Caratozzolo S, Guldolf K, Huhn JI, Loiacono C, Niiberg-Pikksööt T (2019). Understanding the nature of psychiatric comorbidity in migraine: a systematic review focused on interactions and treatment implications. J Headache Pain.

[64] Lampl C, Thomas H, Tassorelli C, Katsarava Z, Laínez JM, Lantéri-Minet M (2016). Headache, depression and anxiety: associations in the Eurolight project. J Headache Pain.

[65] Giri S, Tronvik EA, Hagen K (2022). The bidirectional temporal relationship between headache and affective disorders: longitudinal data from the HUNT studies. J Headache Pain.

[66] Ma M, Zhang J, Chen N, Guo J, Zhang Y, He L (2018). Exploration of intrinsic brain activity in migraine with and without comorbid depression. J Headache Pain.

[67] Ashina S, Bendtsen L, Buse DC, Lyngberg AC, Lipton RB, Jensen R (2017). Neuroticism, depression and pain perception in migraine and tension-type headache. Acta Neurol Scand.

[68] Chu HT, Liang CS, Lee JT, Yeh TC, Lee MS, Sung YF (2018). Associations between depression/anxiety and headache frequency in migraineurs: a cross-sectional study. Headache.

[69] Ligthart L, Gerrits MM, Boomsma DI, Penninx BW (2013). Anxiety and depression are associated with migraine and pain in general: an investigation of the interrelationships. J Pain.

[70] Ashina S, Serrano D, Lipton RB, Maizels M, Manack AN, Turkel CC (2012). Depression and risk of transformation of episodic to chronic migraine. J Headache Pain.

[71] Irimia P, Garrido-Cumbrera M, Santos-Lasaosa S, Aguirre-Vazquez M, Correa-Fernández J, Colomina I (2021). Impact of monthly headache days on anxiety, depression and disability in migraine patients: results from the Spanish Atlas. Sci Rep.

[72] Lee DH, Kim KM, Cho SJ, Kim WJ, Yang KI, Yun CH (2020). Impacts of migraine on the prevalence and clinical presentation of depression: a population-based study. J Affect Disord.

[73] Bigal ME, Lipton RBJHTJoH, Pain F (2008). Concepts and mechanisms of migraine chronification. Headache.

[74] Ornello R, Guerzoni S, Baraldi C, Evangelista L, Frattale I, Marini C (2020). Sustained response to onabotulinumtoxin A in patients with chronic migraine: real-life data. J Headache Pain.

[75] Aaron LA, Burke MM, Buchwald D (2000). Overlapping conditions among patients with chronic fatigue syndrome, fibromyalgia, and temporomandibular disorder. Arch Intern Med.

[76] Nickel JC, Tripp DA, Pontari M, Moldwin R, Mayer R, Carr LK (2010). Interstitial cystitis/painful bladder syndrome and associated medical conditions with an emphasis on irritable bowel syndrome, fibromyalgia and chronic fatigue syndrome. J Urol.

[77] Schäfer I, Kaduszkiewicz H, Wagner H-O, Schön G, Scherer M, van den Bussche H (2014). Reducing complexity: a visualisation of multimorbidity by combining disease clusters and triads. BMC Public Health.

[78] Gelaye B, Sacco S, Brown WJ, Nitchie HL, Ornello R, Peterlin BLJN (2017). Body composition status and the risk of migraine: a meta-analysis. Neurology.

[79] Ornello R, Ripa P, Pistoia F, Degan D, Tiseo C, Carolei A (2015). Migraine and body mass index categories: a systematic review and meta-analysis of observational studies. J Headache Pain.

[80] Bigal ME, Lipton RB, Holland PR, Goadsby PJ (2007). Obesity, migraine, and chronic migraine: possible mechanisms of interaction. Neurology.

[81] Bond DS, O’Leary KC, Thomas JG, Lipton RB, Papandonatos GD, Roth J (2013). Can weight loss improve migraine headaches in obese women? Rationale and design of the Women’s Health and Migraine (WHAM) randomized controlled trial. Contemp Clin Trials.

[82] Bond DS, Thomas JG, Lipton RB, Roth J, Pavlovic JM, Rathier L (2018). Behavioral weight loss intervention for migraine: a randomized controlled trial. Obesity (Silver Spring).

[83] Wang YC, Huang YP, Wang MT, Wang HI, Pan SL (2017). Increased risk of rheumatoid arthritis in patients with migraine: a population-based, propensity score-matched cohort study. Rheumatol Int.

[84] Mathieu S, Couderc M, Pereira B, Dubost J-J, Malochet-Guinamand S, Tournadre A (2020). Prevalence of migraine and neuropathic pain in rheumatic diseases. J Clin Med.

[85] Cloninger CR, Svrakic DM, Przybeck TR (1993). A psychobiological model of temperament and character. Arch Gen Psychiatry.

[86] Garramone F, Baiano C, Russo A, D’Iorio A, Tedeschi G, Trojano L (2020). Personality profile and depression in migraine: a meta-analysis. Neurol Sci.

[87] Costa PT, McCrae RR (1990). Personality disorders and the five-factor model of personality. J Pers Disord.

[88] Breslau N, Chilcoat HD, Andreski P (1996). Further evidence on the link between migraine and neuroticism. Neurology.

[89] Davis RE, Smitherman TA, Baskin SM (2013). Personality traits, personality disorders, and migraine: a review. Neurol Sci.

[90] Holroyd KA, Drew JB, Cottrell CK, Romanek KM, Heh V (2007). Impaired functioning and quality of life in severe migraine: the role of catastrophizing and associated symptoms. Cephalalgia.

[91] Radat F, Lanteri-Minet M, Nachit-Ouinekh F, Massiou H, Lucas C, Pradalier A (2009). The GRIM2005 study of migraine consultation in France. III: Psychological features of subjects with migraine. Cephalalgia.

[92] Bosco MA, Gallinati JL, Clark ME (2013). Conceptualizing and treating comorbid chronic pain and PTSD. Pain Res Treat.

[93] Zale EL, Ditre JW (2015). Pain-related fear, disability, and the fear-avoidance model of chronic pain. Curr Opin Psychol.

[94] Black AK, Fulwiler JC, Smitherman TA (2015). The role of fear of pain in headache. Headache.

[95] Da Silva AN, Lake AE (2014). Clinical aspects of medication overuse headaches. Headache.

[96] Bottiroli S, Galli F, Viana M, De Icco R, Bitetto V, Allena M (2019). Negative short-term outcome of detoxification therapy in chronic migraine with medication overuse headache: role for early life traumatic experiences and recent stressful events. Front Neurol.

[97] Bottiroli S, De Icco R, Vaghi G, Pazzi S, Guaschino E, Allena M (2021). Psychological predictors of negative treatment outcome with Erenumab in chronic migraine: data from an open label long-term prospective study. J Headache Pain.

[98] Raggi A, Giovannetti AM, Leonardi M, Sansone E, Schiavolin S, Curone M (2017). Predictors of 12-months relapse after withdrawal treatment in hospitalized patients with chronic migraine associated with medication overuse: a longitudinal observational study. Headache.

[99] Scaratti C, Covelli V, Guastafierro E, Leonardi M, Grazzi L, Rizzoli PB (2018). A qualitative study on patients with chronic migraine with medication overuse headache: comparing frequent and non-frequent relapsers. Headache.

[100] Winter AC, Berger K, Buring JE, Kurth T (2012). Associations of socioeconomic status with migraine and non-migraine headache. Cephalalgia.

[101] Stewart WF, Staffa J, Lipton RB, Ottman R (1997). Familial risk of migraine: a population-based study. Ann Neurol.

[102] Santos IS, Griep RH, Alves MG, Goulart AC, Lotufo PA, Barreto SM (2014). Job stress is associated with migraine in current workers: the Brazilian Longitudinal Study of Adult Health (ELSA-Brasil). Eur J Pain.

[103] Sandoe CH, Sasikumar S, Lay C, Lawler V (2019). The impact of shift work on migraine: a case series and narrative review. Headache.

[104] Wang Y, Xie J, Yang F, Wu S, Wang H, Zhang X (2015). The prevalence of primary headache disorders and their associated factors among nursing staff in North China. J Headache Pain.

[105] Berry PA (2007). Migraine disorder: workplace implications and solutions. Aaohn J.

[106] Vincent AJ, Spierings EL, Messinger HB (1989). A controlled study of visual symptoms and eye strain factors in chronic headache. Headache.

[107] Lin KC, Huang CC, Wu CC (2007). Association between stress at work and primary headache among nursing staff in Taiwan. Headache.

[108] Linde M, Gustavsson A, Stovner LJ, Steiner TJ, Barré J, Katsarava Z (2012). The cost of headache disorders in Europe: the Eurolight project. Eur J Neurol.

[109] Raggi A, Leonardi M, Sansone E, Curone M, Grazzi L, D’Amico D (2020). The cost and the value of treatment of medication overuse headache in Italy: a longitudinal study based on patient-derived data. Eur J Neurol.

[110] Davies GM, Santanello N, Gerth W, Lerner D, Block GA (1999). Validation of a migraine work and productivity loss questionnaire for use in migraine studies. Cephalalgia.

[111] Reilly MC, Zbrozek AS, Dukes EM (1993). The validity and reproducibility of a work productivity and activity impairment instrument. Pharmacoeconomics.

[112] Raggi A, Covelli V, Guastafierro E, Leonardi M, Scaratti C, Grazzi L (2018). Validation of a self-reported instrument to assess work-related difficulties in patients with migraine: the HEADWORK questionnaire. J Headache Pain.

[113] Al-Hassany L, Haas J, Piccininni M, Kurth T, Maassen Van Den Brink A, Rohmann JL (2020). Giving researchers a headache – sex and gender differences in migraine. Front Neurol.

[114] Sacco S, Ricci S, Degan D, Carolei A (2012). Migraine in women: the role of hormones and their impact on vascular diseases. J Headache Pain.

[115] Hunt K, Adamson J, Hewitt C, Nazareth I (2011). Do women consult more than men? A review of gender and consultation for back pain and headache. J Health Serv Res Policy.

[116] Smitherman TA, Ward TN (2011). Psychosocial factors of relevance to sex and gender studies in headache. Headache.

[117] Adams AM, Serrano D, Buse DC, Reed ML, Marske V, Fanning KM (2015). The impact of chronic migraine: the Chronic Migraine Epidemiology and Outcomes (CaMEO) Study methods and baseline results. Cephalalgia.

[118] Dodick DW, Eross EJ, Parish JM, Silber M (2003). Clinical, anatomical, and physiologic relationship between sleep and headache. Headache.

[119] Coppola G, Di Renzo A, Petolicchio B, Tinelli E, Di Lorenzo C, Serrao M (2020). Increased neural connectivity between the hypothalamus and cortical resting-state functional networks in chronic migraine. J Neurol.

[120] Schulte LH, Allers A, May A (2017). Hypothalamus as a mediator of chronic migraine: evidence from high-resolution fMRI. Neurology.

[121] Moschiano F, Messina P, D’Amico D, Grazzi L, Frediani F, Casucci G (2012). Headache, eating and sleeping behaviors and lifestyle factors in preadolescents and adolescents: preliminary results from an Italian population study. Neurol Sci.

[122] Amin FM, Aristeidou S, Baraldi C, Czapinska-Ciepiela EK, Ariadni DD, Di Lenola D (2018). The association between migraine and physical exercise. J Headache Pain.

[123] Moschiano F, D’Amico D, Ramusino MC, Micieli G (2013). The role of diet and lifestyle in adolescents with headache: a review. Neurol Sci.

[124] Nadelson C (2006). Sport and exercise-induced migraines. Curr Sports Med Rep.

[125] Irby MB, Bond DS, Lipton RB, Nicklas B, Houle TT, Penzien DB (2016). Aerobic exercise for reducing migraine burden: mechanisms, markers, and models of change processes. Headache.

[126] Seok JI, Cho HI, Chung CS (2006). From transformed migraine to episodic migraine: reversion factors. Headache.

[127] Nowaczewska M, Wiciński M, Kaźmierczak W, Kaźmierczak H (2020). To eat or not to eat: a review of the relationship between chocolate and migraines. Nutrients.

[128] Karsan N, Bose P, Newman J, Goadsby PJ (2021). Are some patient-perceived migraine triggers simply early manifestations of the attack?. J Neurol.

[129] Martinelli D, Pocora MM, De Icco R, Putortì A, Tassorelli C (2022). Triggers of migraine: where do we stand?. Curr Opin Neurol.

[130] Rainero I, Govone F, Gai A, Vacca A, Rubino E (2018). Is migraine primarily a metaboloendocrine disorder?. Curr Pain Headache Rep.

[131] Del Moro L, Rota E, Pirovano E, Rainero I (2022). Migraine, brain glucose metabolism and the “neuroenergetic” hypothesis: a scoping review. J Pain.

[132] Di Lorenzo C, Ballerini G, Barbanti P, Bernardini A, D’Arrigo G, Egeo G (2021). Applications of ketogenic diets in patients with headache: clinical recommendations. Nutrients.

[133] Di Lorenzo C, Pinto A, Ienca R, Coppola G, Sirianni G, Di Lorenzo G (2019). A randomized double-blind, cross-over trial of very low-calorie diet in overweight migraine patients: a possible role for ketones?. Nutrients.

[134] Razeghi Jahromi S, Ghorbani Z, Martelletti P, Lampl C, Togha M (2019). Association of diet and headache. J Headache Pain.

[135] Meints SM, Edwards RR (2018). Evaluating psychosocial contributions to chronic pain outcomes. Prog Neuropsychopharmacol Biol Psychiatry.

[136] Afari N, Ahumada SM, Wright LJ, Mostoufi S, Golnari G, Reis V (2014). Psychological trauma and functional somatic syndromes: a systematic review and meta-analysis. Psychosom Med.

[137] Brennenstuhl S, Fuller-Thomson E (2015). The painful legacy of childhood violence: migraine headaches among adult survivors of adverse childhood experiences. Headache.

[138] Fuller-Thomson E, Baker TM, Brennenstuhl S (2010). Investigating the association between childhood physical abuse and migraine. Headache.

[139] Stubberud A, Buse DC, Kristoffersen ES, Linde M, Tronvik E (2021). Is there a causal relationship between stress and migraine? Current evidence and implications for management. J Headache Pain.

[140] Kelman L (2007). The triggers or precipitants of the acute migraine attack. Cephalalgia.

[141] Sauro KM, Becker WJ (2009). The stress and migraine interaction. Headache.

[142] Steiner TJ, Jensen R, Katsarava Z, Linde M, MacGregor EA, Osipova V (2019). Aids to management of headache disorders in primary care (2nd edition): on behalf of the European Headache Federation and lifting the burden: the global campaign against headache. J Headache Pain.

[143] Eigenbrodt AK, Ashina H, Khan S, Diener H-C, Mitsikostas DD, Sinclair AJ (2021). Diagnosis and management of migraine in ten steps. Nat Rev Neur.

[144] Lampl C, Sacco S, Martelletti P (2022). Narrative-based medicine in headache disorders. J Headache Pain.

[145] Heidari E, Rao D, Pfalzgraf AR, Lobo C, Giannetti V (2022). An explorative study of common themes of patient experiences with migraine. Prim Care Companion CNS Disord.

[146] Becker WJ (2002). Communication with the migraine patient. Can J Neurol Sci.

[147] Purdy RA (2002). Migraine: the doctor-patient link. results of a needs assessment. Can J Neurol Sci.

[148] Edmeads J (2002). Communication issues in migraine diagnosis. Can J Neurol Sci.

[149] Buse DC, Lipton RB (2008). Facilitating communication with patients for improved migraine outcomes. Curr Pain Headache Rep.

[150] Ashina M, Buse DC, Ashina H, Pozo-Rosich P, Peres MFP, Lee MJ (2021). Migraine: integrated approaches to clinical management and emerging treatments. Lancet.

[151] Silberstein SD (2015). Preventive migraine treatment. Continuum (Minneapolis, Minn).

[152] American Headache Society (2019) The American Headache Society Position Statement On Integrating New Migraine Treatments Into Clinical Practice. Headache 59:1–18. 10.1111/head.13456 30536394

[153] Steiner TJ (2005). Lifting the burden: the global campaign to reduce the burden of headache worldwide. J Headache Pain.

[154] Klenofsky B, Pace A, Natbony LR, Sheikh HU (2019). Episodic migraine comorbidities: avoiding pitfalls and taking therapeutic opportunities. Curr Pain Headache Rep.

[155] Johnson KG, Ziemba AM, Garb JL (2013). Improvement in headaches with continuous positive airway pressure for obstructive sleep apnea: a retrospective analysis. Headache.

[156] Di Vincenzo A, Beghetto M, Vettor R, Tana C, Rossato M, Bond DS (2020). Effects of surgical and non-surgical weight loss on migraine headache: a systematic review and meta-analysis. Obes Surg.

[157] Verrotti A, Carotenuto M, Altieri L, Parisi P, Tozzi E, Belcastro V (2015). Migraine and obesity: metabolic parameters and response to a weight loss programme. Pediatr Obes.

[158] Peterlin BL (2011). Bariatric surgery in obese migraineurs: mounting evidence but important questions remain. Cephalalgia.

[159] Klonowski T, Kropp P, Straube A, Ruscheweyh R (2022). Psychological factors associated with headache frequency, intensity, and headache-related disability in migraine patients. Neurol Sci.

[160] Nogueira EAG, Oliveira FR, Carvalho VM, Telarolli C, Fragoso YD (2021). Catastrophization is related to the patient and not to the severity of migraine. Arq Neuropsiquiatr.

[161] Patel PS, Minen MT (2019). Complementary and integrative health treatments for migraine. J Neuroophthalmol.

[162] Grazzi L, Sansone E, Rizzoli P (2018). A short review of the non-invasive transcutaneous pericranial electrical stimulation techniques and their application in headache. Curr Pain Headache Rep.

[163] Puledda F, Goadsby PJ (2016). Current approaches to neuromodulation in primary headaches: focus on vagal nerve and sphenopalatine ganglion stimulation. Curr Pain Headache Rep.

[164] Shirahige L, Melo L, Nogueira F, Rocha S, Monte-Silva K (2016). Efficacy of noninvasive brain stimulation on pain control in migraine patients: a systematic review and meta-analysis. Headache.

[165] Martelletti P, Jensen RH, Antal A, Arcioni R, Brighina F, de Tommaso M (2013). Neuromodulation of chronic headaches: position statement from the European Headache Federation. J Headache Pain.

[166] Pérez-Muñoz A, Buse DC, Andrasik F (2019). Behavioral Interventions for Migraine. Neurol Clin.

[167] Lee HJ, Lee JH, Cho EY, Kim SM, Yoon S (2019). Efficacy of psychological treatment for headache disorder: a systematic review and meta-analysis. J Headache Pain.

[168] Faedda N, Natalucci G, Baglioni V, Giannotti F, Cerutti R, Guidetti V (2019). Behavioral therapies in headache: focus on mindfulness and cognitive behavioral therapy in children and adolescents. Expert Rev Neurother.

[169] Raggi A, Grignani E, Leonardi M, Andrasik F, Sansone E, Grazzi L (2018). Behavioral approaches for primary headaches: recent advances. Headache.

[170] Andrasik F, Grazzi L, D’Amico D, Sansone E, Leonardi M, Raggi A (2016). Mindfulness and headache: a “new” old treatment, with new findings. Cephalalgia.

[171] Orr SL (2016). Diet and nutraceutical interventions for headache management: a review of the evidence. Cephalalgia.

[172] Rajapakse T, Pringsheim T (2016). Nutraceuticals in migraine: a summary of existing guidelines for use. Headache.

[173] Orr SL, Venkateswaran S (2014). Nutraceuticals in the prophylaxis of pediatric migraine: evidence-based review and recommendations. Cephalalgia.

[174] Adams J, Barbery G, Lui CW (2013). Complementary and alternative medicine use for headache and migraine: a critical review of the literature. Headache.

[175] Grazzi L, Toppo C, D’Amico D, Leonardi M, Martelletti P, Raggi A (2021). Non-pharmacological approaches to headaches: non-invasive neuromodulation, nutraceuticals, and behavioral approaches. Int J Environ Res Public Health.

[176] Ng JY, Hanna C (2021). Headache and migraine clinical practice guidelines: a systematic review and assessment of complementary and alternative medicine recommendations. BMC Complem Med Ther.

[177] Onofri A, Necozione S, Tozzi E (2020). Complementary and alternative medicine (CAM) in headache of children and adolescents: open-label Italian study. Clin Ter.

[178] Zhang Y, Dennis JA, Leach MJ, Bishop FL, Cramer H, Chung VCH (2017). Complementary and alternative medicine use among US adults with headache or migraine: results from the 2012 National Health Interview Survey. Headache.

[179] Coppola G, Di Lorenzo C, Serrao M, Parisi V, Schoenen J, Pierelli F (2016). Pathophysiological targets for non-pharmacological treatment of migraine. Cephalalgia.

[180] Chae JS, Paik JK, Kang R, Kim M, Choi Y, Lee SH (2013). Mild weight loss reduces inflammatory cytokines, leukocyte count, and oxidative stress in overweight and moderately obese participants treated for 3 years with dietary modification. Nutr Res.

[181] Seminowicz DA, Burrowes SAB, Kearson A, Zhang J, Krimmel SR, Samawi L (2020). Enhanced mindfulness-based stress reduction in episodic migraine: a randomized clinical trial with magnetic resonance imaging outcomes. Pain.

[182] Dhand A, Luke DA, Lang CE, Lee JM (2016). Social networks and neurological illness. Nat Rev Neurol.

[183] Sacco S, Lampl C, Maassen van den Brink A, Caponnetto V, Braschinsky M, Ducros A (2021). Burden and attitude to resistant and refractory migraine: a survey from the European Headache Federation with the endorsement of the European Migraine & Headache Alliance. J Headache Pain.

[184] Diener HC, Antonaci F, Braschinsky M, Evers S, Jensen R, Lainez M (2020). European Academy of Neurology guideline on the management of medication-overuse headache. Eur J Neurol.

[185] Gaul C, Visscher CM, Bhola R, Sorbi MJ, Galli F, Rasmussen AV (2011). Team players against headache: multidisciplinary treatment of primary headaches and medication overuse headache. J Headache Pain.

[186] Colloca L, Barsky AJ (2020). Placebo and Nocebo Effects. New Eng J Med.

[187] Forbes RB, McCarron M, Cardwell CR (2020). Efficacy and contextual (Placebo) effects of CGRP Antibodies for migraine: systematic review and meta-analysis. Headache.

[188] Loder EW, McGeeney B (2020). Disentangling placebo effects in the treatment of migraine. Nat Rev Neur.

[189] Powers SW, Coffey CS, Chamberlin LA, Ecklund DJ, Klingner EA, Yankey JW (2016). Trial of amitriptyline, topiramate, and placebo for pediatric migraine. New Eng J Med.

[190] McLaren NJA, Psychiatry NZJo (1998). A critical review of the biopsychosocial model. Aust N Z J Psychiatry.

[191] Bolton D, Gillett G (2019). The biopsychosocial model 40 years on.

[192] Kontos NJAM (2011). Perspective: biomedicine—menace or straw man? Reexamining the biopsychosocial argument. Acad Med.

[193] Cormack B, Stilwell P, Coninx S, Gibson J (2022) The biopsychosocial model is lost in translation: from misrepresentation to an enactive modernization. Physiother Theory Prac 8:1–16 10.1080/09593985.2022.2080130 35645164

[194] Steiner TJ, Jensen R, Katsarava Z, Stovner LJ, Uluduz D, Adarmouch L (2021). Structured headache services as the solution to the ill-health burden of headache: 1 Rationale and description. J Headache Pain.

[195] Tinelli M, Leonardi M, Paemeleire K, Raggi A, Mitsikostas D, de la Torre ER (2021). Structured headache services as the solution to the ill-health burden of headache. 3. Modelling effectiveness and cost-effectiveness of implementation in Europe: findings and conclusions. J Headache Pain.

[196] Tinelli M, Leonardi M, Paemeleire K, Mitsikostas D, de la Torre ER, Steiner TJ (2021). Structured headache services as the solution to the ill-health burden of headache. 2. Modelling effectiveness and cost-effectiveness of implementation in Europe: methodology. J Headache Pain.

[197] Ashina M, Terwindt GM, Al-Karagholi MA, de Boer I, Lee MJ, Hay DL (2021). Migraine: disease characterisation, biomarkers, and precision medicine. Lancet.

[198] Schork NJ (2015). Personalized medicine: time for one-person trials. Nature.

