# Correction: Applying a biopsychosocial model to migraine: rationale and clinical implications

**DOI:** 10.1186/s10194-022-01487-9

**Published:** 2022-09-07

**Authors:** Chiara Rosignoli, Rafaele Ornello, Agnese Onofri, Valeria Caponnetto, Licia Grazzi, Alberto Raggi, Matilde Leonardi, Simona Sacco

**Affiliations:** 1 Department of Biotechnological and Applied Clinical Sciences, University of L’Aquila, L’Aquila, Italy; 2 Neuroalgology Unit and Headache Centre, Fondazione IRCCS Istituto Neurologico Carlo Besta, Milan, Italy; 3 Neurology, Public Health and Disability Unit, Fondazione IRCCS Istituto Neurologico Carlo Besta, Milan, Italy

**Correction: J Headache Pain 23, 100 (2022)**

**https://doi.org/10.1186/s10194-022-01471-3**

Following the publication of the original article [1], we were notified that the arrows displayed in Fig. 4 were misaligned in the pdf version of the paper.

The original article has been corrected.
